# Differential Pro-Inflammatory Responses of Astrocytes and Microglia Involve STAT3 Activation in Response to 1800 MHz Radiofrequency Fields

**DOI:** 10.1371/journal.pone.0108318

**Published:** 2014-10-02

**Authors:** Yonghui Lu, Mindi He, Yang Zhang, Shangcheng Xu, Lei Zhang, Yue He, Chunhai Chen, Chuan Liu, Huifeng Pi, Zhengping Yu, Zhou Zhou

**Affiliations:** 1 Department of Occupational Health, Key Lab of Medical Protection for Electromagnetic Radiation, Ministry of Education of China, Third Military Medical University, Chongqing, China; 2 Department of Laboratory Medicine, Southwest Hospital, Third Military Medical University, Chongqing, China; 3 Department of Neurosurgery, Tongji Hospital, Tongji Medical College, Huazhong University of Science and Technology, Wuhan, China; Indiana School of Medicine, United States of America

## Abstract

Microglia and astrocytes play important role in maintaining the homeostasis of central nervous system (CNS). Several CNS impacts have been postulated to be associated with radiofrequency (RF) electromagnetic fields exposure. Given the important role of inflammation in neural physiopathologic processes, we investigated the pro-inflammatory responses of microglia and astrocytes and the involved mechanism in response to RF fields. Microglial N9 and astroglial C8-D1A cells were exposed to 1800 MHz RF for different time with or without pretreatment with STAT3 inhibitor. Microglia and astrocytes were activated by RF exposure indicated by up-regulated CD11b and glial fibrillary acidic protein (GFAP). However, RF exposure induced differential pro-inflammatory responses in astrocytes and microglia, characterized by different expression and release profiles of IL-1β, TNF-α, IL-6, PGE2, nitric oxide (NO), inducible nitric oxide synthase (iNOS) and cyclooxygenase 2 (COX2). Moreover, the RF exposure activated STAT3 in microglia but not in astrocytes. Furthermore, the STAT3 inhibitor Stattic ameliorated the RF-induced release of pro-inflammatory cytokines in microglia but not in astrocytes. Our results demonstrated that RF exposure differentially induced pro-inflammatory responses in microglia and astrocytes, which involved differential activation of STAT3 in microglia and astrocytes. Our data provide novel insights into the potential mechanisms of the reported CNS impacts associated with mobile phone use and present STAT3 as a promising target to protect humans against increasing RF exposure.

## Introduction

The growing use of mobile phones worldwide has provoked public concern regarding the potential risks of radiofrequency (RF) electromagnetic fields to the central nervous system (CNS). Based on experimental data, RF exposure has been postulated to cause a variety of neurological effects. Several studies have reported that exposure to RF fields could induce headache [1], neuron and glial cell death [2], [3], disturbances in neurotransmitter release [4] and cognitive impairments [5], and increase blood-brain barrier permeability [6]. Although there are lots of positive reports, it is still controversial whether RF exposure leads to these CNS impacts. Some of these effects were not observed by some other studies after RF exposure [7]–[10]. However, long-term exposure to mobile phone RF fields has been reported to increase the risk for neuroma and glioma [11]. Recently, RF electromagnetic fields has been classified as possibly carcinogenic to humans (Group 2B) by the International Agency for Research on Cancer (IARC) [12]. In addition, although the clinical significance is not clear, a recent study demonstrated that brain glucose metabolism was changed by cell phone signals [13]. However, the underlying reasons of these induced CNS disorders are largely unknown.

Given the important role of glial cells in maintaining the homeostasis of the CNS, several studies have focused on the activation of microglia and astrocytes after RF exposure with different frequencies. The astrocyte has been reported to be activated by exposure to 835 and 900 MHz RF [14], [15] and microglia activation was observed after exposure to 915 and 2450 MHz RF [16], [17] along with enhanced pro-inflammatory cytokines. Inconsistently, some other studies did not reveal alterations in microglia and astrocyte activations after 900 and 1950 MHz RF exposure [18], [19]. Despite the conclusions are not coincident, the mechanisms of these cellular activations are not understood. To our knowledge, very few studies have investigated the pro-inflammation responses and the involved mechanisms of microglia and astrocytes after exposure to GSM (Global System for Mobile Communication) 1800 MHz RF, which is widely used in Europe and China.

Microglia are the innate immune cells and perform a constitutive surveillance role in the CNS. Microglial cells are activated in response to a variety of stimuli and pathological events such as brain injury, infection, neurodegeneration and brain tumors and play important pathophysiological roles. Activation endows microglia with a reactive profile characterized by morphological, immunophenotypical and functional alterations [20], [21]. Over-activated microglia will undergo a pro-inflammatory response by releasing inflammatory cytokines such as interleukin-1 beta (IL-1β), interleukin-6 (IL-6), Chemokine (C-C motif) ligand 2 (CCL2) and tumor necrosis factor-α (TNF-α) [20], [22]. Two inducible syntheses, inducible nitric oxide synthase (iNOS) and cyclooxygenase 2 (COX2), can be up-regulated upon microglia activation, producing two pro-inflammatory cytokines of nitric oxide (NO) and prostaglandin E2 (PGE2) [23], [24].

Astrocytes are the major glial cell in the brain, performing structural and metabolic functions. Furthermore, astrocyte responses to diverse pathological CNS insults, such as traumatic injury, ischemia, neurotoxic chemicals, tumors, infections and neurodegenerative diseases, are characterized by variations in morphology and molecular expression pattern [25]. In response to CNS insults, activated astrocytes can express and release pro-inflammatory cytokines that will lead to neuroinflammation, including IL-1β, IL-6, TNF-α and CCL2 [26]–[28]. Additionally, iNOS and COX2 expressions may increase in activated astrocytes, resulting in neuroinflammation by the production of the cytokines NO and PGE2 [29], [30].

Signal transducer and activator of transcription 3 (STAT3) is an important regulator of inflammatory gene expression. STAT3 has been also shown to mediate pro-inflammatory responses in microglia and astrocytes in response to various CNS insults. It has been reported that STAT3 was activated and driven expression of inflammatory cytokines in both microglia and astrocytes [31], [32]. Our previous studies have demonstrated that microglial cells are activated by 2.45 GHz electromagnetic fields, which involves STAT3 activation [17]. However, whether 1800 MHz RF activates STAT3 in microglia and whether STAT3 is involved in the RF-induced pro-inflammatory responses in astrocytes has not been studied.

The present study reveals that exposure to 1800 MHz RF induced activation of microglia and astrocytes, but differentially triggered the expression and release of pro-inflammatory cytokines in cultured microglia and astrocytes. This study demonstrates for the first time that STAT3 was activated and involved in 1800 MHz RF stimulated microglia but not in astrocytes, which might mediated the differential pro-inflammatory responses induced by RF exposure in microglia and astrocytes.

## Materials and Methods

### Cell culture

Mouse microglial cells (N9) were kindly provided by Dr. Bai Yun (Department of Genetics, The Third Military Medical University, China). The N9 microglia was obtained by immortalization of E13 mouse embryonic brain cultures with the 3RV retrovirus carrying an activated v-myc oncogene [33] and shares many phenotypical characteristics with primary mouse microglia [34]. N9 cells were maintained in Iscove's modified Dulbecco's medium (IMDM) (HyClone, Logan, UT) supplemented with 5% heat-inactivated fetal bovine serum (HyClone), 2 mM glutamine, 100 µg/mL streptomycin, 100 U/mL penicillin (Beyotime, Jiangsu, China) and 50 µM 2-mercaptoethanol (Sigma-Aldrich, St. Louis, MO, USA). Mouse astrocyte type I cells (C8-D1A) (ATCC, Rockville, MD, USA) were cultured in Dulbecco's Modified Eagle's medium (DMEM) (GIBCO, Gran Island, NY, USA). Culture medium was supplemented with 10% fetal bovine serum, 100 U/mL penicillin and 100 µg/mL streptomycin (Beyotime). Both microglial and astroglial cells were plated in 25-cm^2^ T-flasks (Corning, Tewksbury, MA, USA) and incubated at 37°C in a humid atmosphere with 5% CO_2_.

### RF exposure system

The standard RF exposure system (sXc1800) was commercially provided by the Foundation for Information Technologies in Society (IT'IS Foundation, Zurich, Switzerland). A detailed description, including evaluations of specific absorption rate (SAR), distribution of SAR, temperature load during RF exposure, dielectric constants and ambient electric fields, was put forth by the designer [35]. Briefly, the sXc1800 system consists of two resonating R18-waveguides, a RF signal generator, a RF power amplifier, a function generator, a data acquisition unit and a digital control unit. In each waveguide chamber, a Petri dish holder for six 35 mm Petri dishes ensures that each dish is positioned accurately in the H-field maximum of the standing wave. To avoid the potential thermal effect of RF fields, two ventilation holes are located on the waveguide and a ventilator fan is fixed to one hole to cool the culture medium. The temperature is monitored by a field detector and the temperature rise is less than 0.05°C under our exposure setting. The field is monitored by a field detector. Both waveguides are placed in an incubator to ensure constant environmental conditions (37±0.2°C, 5% CO2, 95% air atmosphere). The RF generator is modulated by the function generator, and then the signal is amplified by the RF power amplifier. The system and exposure, including exposure strength (SAR), time and pattern, are automatically controlled by a computer.

### Cell treatment procedure

Both microglial and astroglial cells were sub-cultured by plating 5×10^5^ cells per 35 mm Petri dish (Corning) before RF exposure. Twenty four hours after seeding, the cells were divided into sham groups and exposure groups, and were subjected to RF exposure or sham exposure for 1, 3, 6, 12 and 24 hours. The cells were exposed to GSM 1800 MHz RF modulated by a rectangular pulse with a repetition frequency of 217 Hz at an average SAR of 2.0 W/kg with an intermittent duration of 5 min on and 10 min off. The computer randomly assigned the exposure waveguide and sham waveguide, achieving blind experiments, and then the cells received RF exposure or sham exposure simultaneously. There were no RF signals in the sham waveguide. After exposure, cells and cultured supernatants were harvested and subjected to subsequent tests. In addition, naive control groups and lipopolysaccharide (LPS) control groups were set up for each time-point. The naive controls did not receive any treatments and the LPS groups were treated with 1 µg/mL LPS. The STAT3 inhibitor Stattic (Santa Cruz Biotechnology, Texas, U.S.A.) pretreatments were carried out by administration of 20 µmol/L Stattic 45 min prior to 24-h RF exposure.

### Flow cytometry analysis

Fluorescence-activated cell sorting (FACS) analysis was used to determine the expression of microglial marker CD11b. After RF exposure and sham exposure, microglial cells were harvested in 1.5 mL centrifuge tubes. The cells were washed three times with staining buffer (phosphate buffered saline (PBS) containing 0.1% sodium azide and 1% bovine serum albumin (BSA)) and incubated with goat serum (Zhongshan Goldenbridge Biotechnology, Beijing, China) for 20 min at 4°C. Then, the cells were incubated with rat anti-mouse monoclonal antibody CD11b (1∶100; AbD Serotec, Oxford, UK) or rat IgG2b isotype control (1∶100; AbD Serotec) for 30 min at 4°C. Following three washes with staining buffer, the cells were then incubated with fluorescently labeled rabbit anti-rat IgG (1∶100; Invitrogen, Carlsbad, CA, USA) for 30 min at 4°C in the dark. After the final wash, cells were fixed with 2% paraformaldehyde and analyzed using a flow cytometer (BD Biosciences, San Jose, CA). Three independent experiments were performed.

### Immunocytochemistry

We performed the immunocytochemistry to detect GFAP in astrocytes after the RF exposure. Astrocytes were plated on glass coverslips and subjected to RF exposure in the Petri dishes as above-mentioned. After RF exposure and sham exposure, the cells were fixed with 4% paraformaldehyde for 15 min at room temperature. After washing, the cells were permeabilized with 0.3% TritonX-100 for 10 min at room temperature. Following three 5-min washes in PBS, the cells were blocked with 1% BSA at room temperature for 30 min. The cells were then incubated with rabbit primary antibody against GFAP (1∶200, Abcam, Cambridge, UK) overnight at 4°C. The following day, the cells were washed three times with PBS, and then incubated with fluorescently labeled chicken anti-rabbit IgG (1∶100, Invitrogen) for 1 h at room temperature in the dark. Nuclear staining of the fixed cells was performed with Hoechst 33342 (0.5 µg/mL, Sigma-Aldrich). The fluorescence images of the stained cells were captured using a confocal laser-scanning microscope (ZEISS, Germany). The experiment was executed in triplicate.

### Western blotting

To determine the protein levels and phosphorylation, the immunoblotting was performed. After RF exposure and sham exposure, the microglial and astroglial cells were lysed using lysis buffer (Beyotime) containing a cocktail of protease inhibitors (Sigma-Aldrich). The total protein concentration (µg/µL) was measured by a BCA protein assay kit (Beyotime) using BSA as a standard. Equal amounts of proteins (40 µg/lane) were separated by 8% or 10% sodium dodecyl sulfate polyacrylamide gel electrophoresis (SDS-PAGE) and subsequently transferred to a nitrocellulose membrane (Millipore Corp., Bedford, MA, USA). After blocking in 5% dried skimmed milk in PBS, the membranes were incubated with primary antibodies: rabbit anti-iNOS (Abcam), rabbit anti-COX2 (Abcam), rabbit anti-GFAP (Abcam), rabbit anti-phospho-STAT3 (Cell Signal Technology, Danvers, USA), mouse anti-STAT3 (Cell Signal Technology) and mouse anti-β-actin (Sigma-Aldrich). Membranes were then incubated in fluorescently labeled secondary antibodies (LI-COR, Lincoln, USA). The fluorescence signals were detected and quantified with the Odyssey infrared imaging system (LI-COR). For each protein, three independent immumoblots were carried out.

### Real-time reverse transcription-PCR (RT-PCR)

The gene expressions of pro-inflammatory cytokines and proteins were measured by real-time PCR. Total RNA was extracted from the exposed or sham-exposed cells by using RNAiso Plus (TaKaRa Biotechnology, Dalian, China) according to the manufacturer's instructions. The RNA concentration (ng/µL) was detected using a spectrophotometer (Thermo Scientific, Waltham, USA). Then, 1 µg of total RNA was reverse-transcribed at 37°C for 15 min using a reverse transcription kit (TaKaRa). The cDNA products were subsequently subjected to real-time PCR analysis using a real-time PCR kit (TaKaRa) with a real-time PCR system (Bio-Rad, Hercules, USA). The amplification of cDNA was performed with the following primers: IL-1β: forward 5′-TGGTGTGTGACGTTCCCATTA-3′, reverse 5′-CAGCACGAGGCTTTTTTGTTG-3′; IL-6: forward 5′-ACAACCACGGCCTTCCCTACTT-3′, reverse 5′-CACGATTTCCCAGAGAACATGTG-3′; TNF-α: forward 5′-GGCAGGTCTACTTTGGAGTCATTG-3′, reverse 5′-ACATTCGAGGCTCCAGTGAATTCGG-3′; CCL2: forward 5′-AGCACCAGCCAACTCTCACT-3′, reverse 5′-CGTTAACTGCATCTGGCTGA-3′ and Glyceraldehyde-3-phosphate dehydrogenase (GAPDH): forward 5′-AGGTCGGTGTGAACGGATT-3′, reverse 5′-AATCTCCACTTTGCCACTGC-3′. The reaction mixtures were initially denatured at 95°C for 30 s, followed by 40 amplification cycles: 95°C, 5 s; 60°C, 30 s. For each gene, three independent experiments were performed.

### Enzyme-linked immunosorbent assay (ELISA)

The releases of pro-inflammatory cytokines in the culture medium were detected by ELISA. Levels of IL-1β, IL-6, TNF-α and PGE2 in the cultured supernatants after RF exposure were measured using IL1 beta Mouse ELISA Kit (Abcam), IL-6 Mouse ELISA Kit (Abcam), Mouse Prostaglandin E2 (PG-E2) ELISA Kit (CUSABIO, Wuhan, China), Mouse TNF-α ELISA Kit (BioLegend, San Diego, USA) and ELISA Kit for Tumor Necrosis Factor Alpha (TNFa) (Uscn Life Science Inc., Wuhan, China), according to the manufacturers' protocols.

### Nitric oxide (NO) measurement

NO release was measured by detecting NO metabolites (nitrates and nitrites) in the cell culture supernatant using a Total NO Assay Kit (Beyotime). Briefly, 50 µL standard or 50 µL cell culture supernatant was added to each well. Then, 50 µL of nicotinamide adenine dinucleotide (NADH) and nitrate reductase was added. After 30 min, 50 µL of Greiss reagents I and 50 µL Griess reagent II were added and incubated for 10 min at room temperature. The absorbance of each well was measured at 540 nm with a microplate reader (TECAN, Switzerland) and converted to nitrite concentration (µM). All samples were tested in triplicate and three independent experiments were performed.

### Electrophoresis mobility shift assay (EMSA)

To test the binding of STAT3 to DNA, a Chemiluminescent EMSA Kit (Beyotime) was used following the manufacturer's instruction. Cell nuclear proteins were extracted using a nuclear protein extract kit (Beyotime) according to the manufacturer's protocol. The following oligonucleotide sequences were used as the biotin-labeled STAT3 consensus oligonucleotide probes and cold competitors: 5′-GAT CCT TCT GGG AAT TCC TAG ATC-3′, 3′-CTA GGA AGA CCC TTA AGG ATC TAG-5′. The mixtures containing 10 µg nuclear extract proteins and STAT3 probes were incubated for 20 min at room temperature. Electrophoresis was performed at 10 V/cm using 6% native polyacrylamide gels and then shifted samples were transferred to a positively charged nitrocellulose membrane. The gel shifts were visualized with a gel imaging system (Bio-Rad). Three independent experiments were carried out.

### Statistical analysis

The data are presented as the mean ± SEM. All the statistical comparisons were performed by SPSS 18.0 (SPSS Inc., Chicago, IL, USA). The data were compared using unpaired Student's t-test and one-way analysis of variance (ANOVA), followed by Newman-Keuls multiple comparison test. A value of p<0.05 was considered to be statistically significant.

## Results

### Activations of microglia and astrocytes after RF exposure

**Figure 1:**
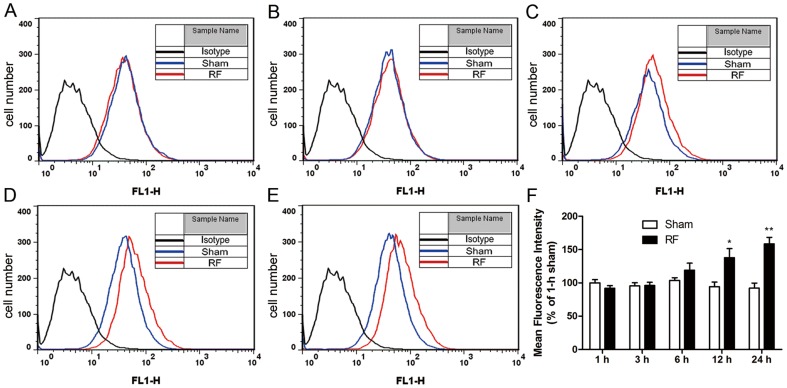
CD11b expression following exposure to RF on microglial N9 cell surface. N9 cells suffered sham exposure (0 W/kg) or 1800 MHz RF exposure (2 W/kg) for 1 h (A), 3 h (B), 6 h (C), 12 h (D) and 24 h (E), respectively. CD11b fluorescence intensity was measured by FACS, rat IgG2b was used as isotype control. (A-E) Fluorescence intensity curves of one experiment presented by time-points. (F) Mean fluorescence intensities ± SEM of CD11b from three independent experiments are expressed as the percentage of sham exposure for 1 h. ^*^p<0.05, ^**^p<0.01 compared with the corresponding sham groups.

**Figure 2:**
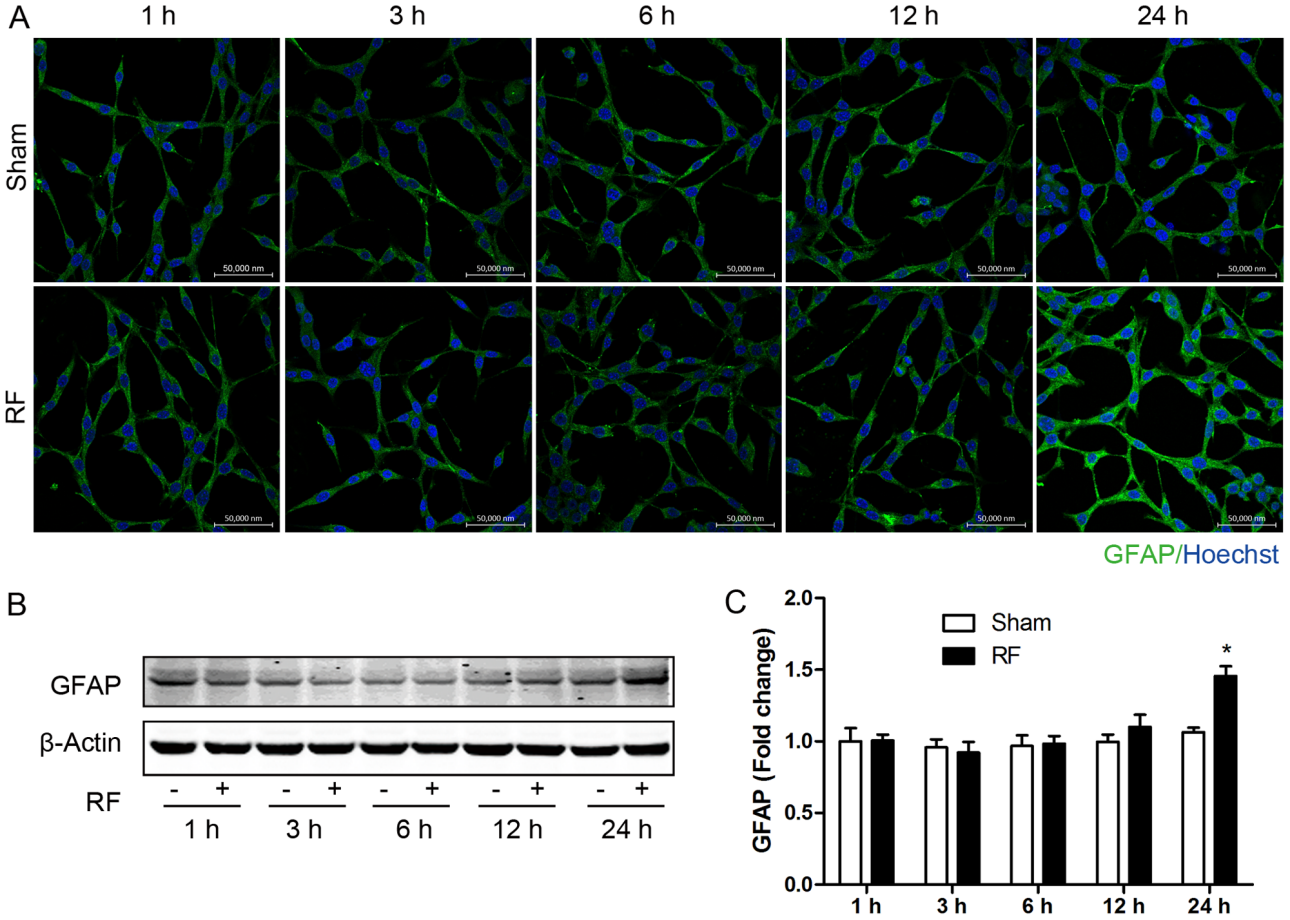
GFAP immunoreactivity and expression following exposure to RF in astroglial C8-D1A cells. (A) Immunofluorescence staining of GFAP (green) and nuclear staining (blue) in C8-D1A cells, scale bar 50 µm. (B) Immunoblotting of GFAP after sham and RF exposure for 1, 3, 6, 12 and 24 h. (C) Densitometric analysis of GFAP immunoblotting as normalized to 1-h sham control. All experiments were performed in triplicate and error bars represent mean ± SEM. ^*^p<0.05 compared with corresponding sham groups.

We firstly assessed CD11b expression on the surface of N9 cells to investigate the effects of RF exposure on microglia activation. Flow cytometry results showed that CD11b expression on surface of N9 cells increased after exposure to RF for 12 and 24 h compared with sham control (Figure 1A-F). The responses of astrocytes upon RF exposure were analyzed by means of the expression of glial fibrillary acidic protein (GFAP) applying confocal microscopy and immunoblotting. Immunoreactivity of GFAP was enhanced by 24-h RF exposure in astrocytes (Figure 2A), and it was further verified by western blot, showing that GFAP expression increased after exposure to RF for 24 h (Figure 2B, C).

### RF exposure differentially induced gene expressions of pro-inflammatory cytokines in microglia and astrocytes

**Figure 3:**
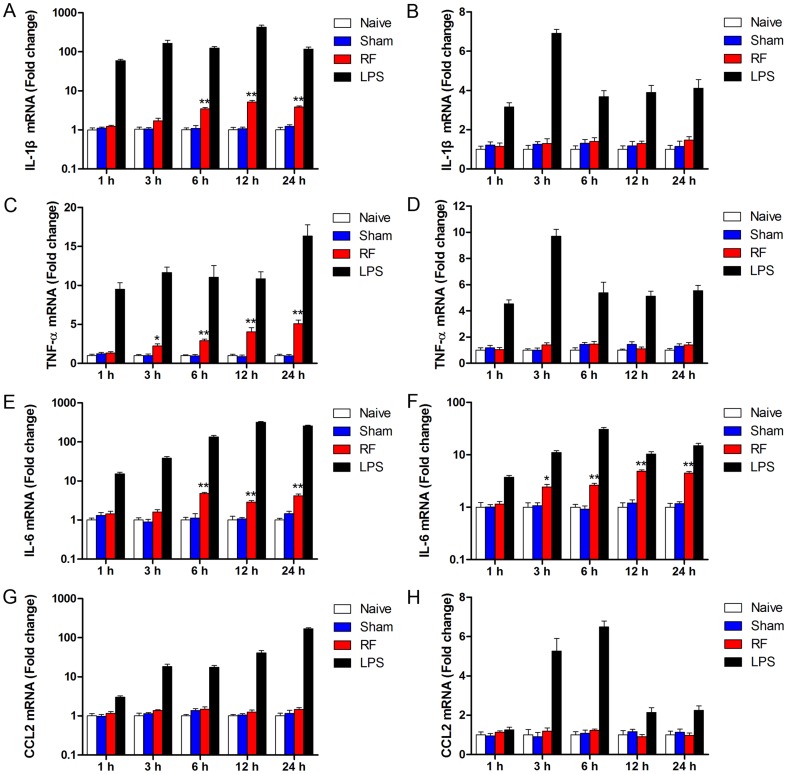
Differential gene expressions of cytokines induced by RF exposure in microglia and astrocytes. mRNA levels of IL-1β, TNF-α, IL-6 and CCL2 were determined by real-time PCR in microglia (A, C, E, G) and astrocytes (B, D, F, H). Naive controls (no treatment) and LPS controls (1 µg/mL) were set up at each point. Values are mean ± SEM for three independent experiments and are expressed as fold increase compared to naive controls at each point. ^*^p<0.05, ^**^p<0.01 compared with corresponding sham groups.

As mentioned above, the cytokines IL-1β, TNF-α, IL-6 and CCL2 were up-regulated and involved in the inflammation progress after the activation of astrocytes and microglia. To investigate the effect of RF exposure on gene expression of the cytokines, mRNA levels of IL-1β, TNF-α, IL-6 and CCL2 were determined after RF exposure in astrocytes and microglia. In microglia, RT-PCR analysis revealed that mRNA levels of IL-1β increased after 6-h RF exposure and kept at 12 and 24 h (Figure 3A). TNF-α gene expression was enhanced by as early as 3-h RF exposure, which maintained at 6, 12 and 24 h in microglia (Figure 3C). However, RF exposure did not affect gene expression of IL-1β and TNF-α at any time-point in astrocytes (Figure 3B, D). IL-6 mRNA levels increased after 6-h RF exposure in microglia (Figure 3E) and 3-h RF exposure in astrocytes (Figure 3F), respectively. mRNA expression of CCL2 was not affected by RF exposure in both microglia and astrocytes (Figure 3G, H). In addition, naive controls that did not receive any treatment and positive controls stimulated by LPS were set up at each time point. As shown in Figure 3, no significant differences in cytokine expression were observed between the naive and sham groups and LPS treatment led to a continuous increase of cytokine expression in microglia and astrocytes.

### RF exposure differentially induced cytokine releases in microglia and astrocytes

**Figure 4:**
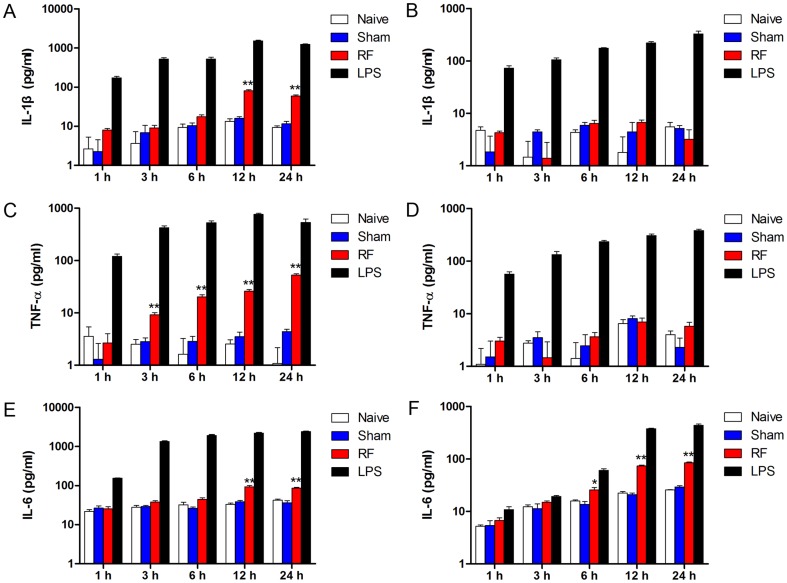
RF exposure differentially induced cytokine release in microglia and astrocytes. The supernatants were collected and used for an ELISA test after sham and RF exposure. Naive controls (no treatment) and LPS controls (1 µg/mL) were set up at each point. (A, C, E) Releases of IL-1β, TNF-α and IL-6 upon sham, RF and LPS treatments in microglial N9 cells. (B, D, F) Releases of IL-1β, TNF-α and IL-6 upon sham, RF and LPS treatments in astroglial C8-D1A cells. Values are mean ± SEM for three independent experiments. ^*^p<0.05, ^**^p<0.01 compared with corresponding sham groups.

Given that RF exposure induced mRNA expression of pro-inflammatory cytokines, we analyzed the release of IL-1β, TNF-α and IL-6 in cell culture supernatants after RF exposure for different time. In microglia, IL-1β release was significantly elevated by 12 and 24-h RF exposure compared with sham controls (Figure 4A). Nevertheless, RF exposure did not alter the release of IL-1β at all studied time-points in astrocytes (Figure 4B). No differences in IL-1β levels between naive and sham controls were observed and LPS treatment led to a continuous increase of IL-1β in both microglia and astrocytes (Figure 4A, B). TNF-α production increased after exposure to RF for 3, 6, 12 and 24 h compared with sham controls in microglia (Figure 4C). In contrast, RF exposure did not induce alterations of TNF-α release in astrocytes (Figure 4D). In both microglia and astrocytes, sham exposure did not affect TNF-α release, which increased upon LPS treatment at all studied time-points (Figure 4C, D). However, RF exposure caused a significant increase of IL-6 release after exposure to RF for 6 h in astrocytes and 12 h in microglia (Figure 4E, F). Additionally, sham exposure did not affect IL-6 release, which increased upon exposure to control LPS.

### RF exposure differentially induced expression of iNOS and COX2, and production of NO and PGE2 in microglia and astrocytes

**Figure 5:**
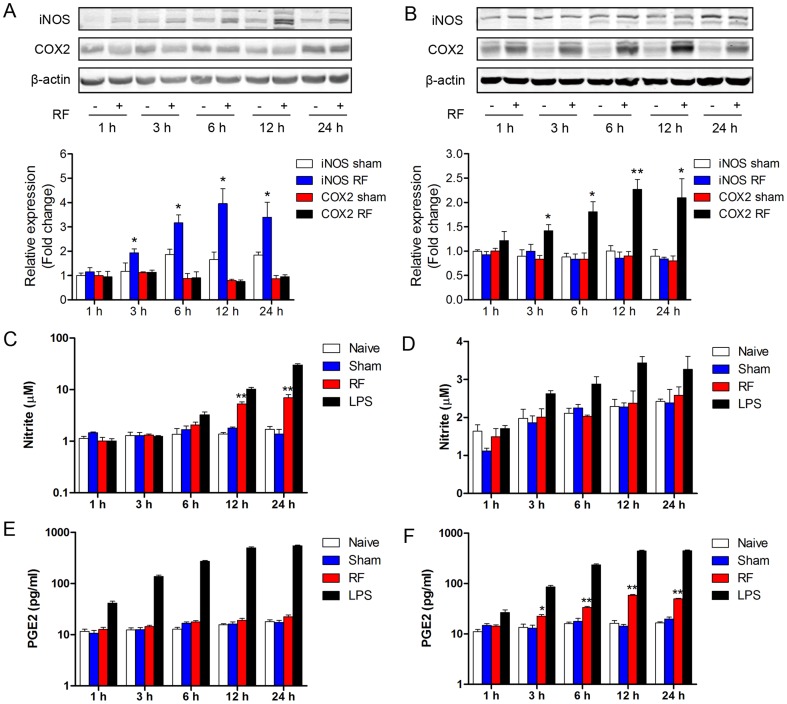
Levels of iNOS and COX2 and their production following RF exposure in microglia and astrocytes. Western blot, Griess reaction and ELISA assays were performed to determined iNOS and COX2 protein levels, NO production and PGE2 release in microglial N9 cells (A, C, E) and astroglial C8-D1A cells (B, D, F). Naive controls (no treatment) and LPS controls (1 µg/mL) were set up at each point. Values are mean ± SEM for three independent experiments. ^*^p<0.05, ^**^p<0.01 compared with corresponding sham groups.

We further investigated the expression of iNOS and COX2, and the resulting NO and PGE2 production after exposure to RF. The western blotting results showed that iNOS protein expression was enhanced by 3, 6, 12 and 24-h RF exposure in microglia (Figure 5A). Correspondingly, microglial NO production was significantly augmented upon RF exposure for 12 and 24 h (Figure 5C). Whereas the RF exposure did not alter the protein expression of iNOS at all studied time-points in astrocytes (Figure 5B). Moreover, the level of NO did not change after RF exposure in astrocytes (Figure 5D).

Differently from the iNOS expression pattern induced by RF exposure, the protein expressions of COX2 were not affected by RF exposure at all indicated time-points in microglia (Figure 5A). Correspondingly, the RF exposure did not induce significant alterations in the release of microglial PGE2 (Figure 5E). In contrast, the western blotting analysis confirmed that RF exposure induced COX2 protein expression after 3-h RF exposure in astrocytes (Figure 5B). We further determined the release of PGE2, which is the product of inducible COX2, demonstrating that PGE2 production increased upon RF exposure for 3, 6, 12 and 24 h (Figure 5F).

### Phosphorylation and DNA-binding activity of STAT3 were differentially regulated by RF exposure in microglia and astrocytes

**Figure 6:**
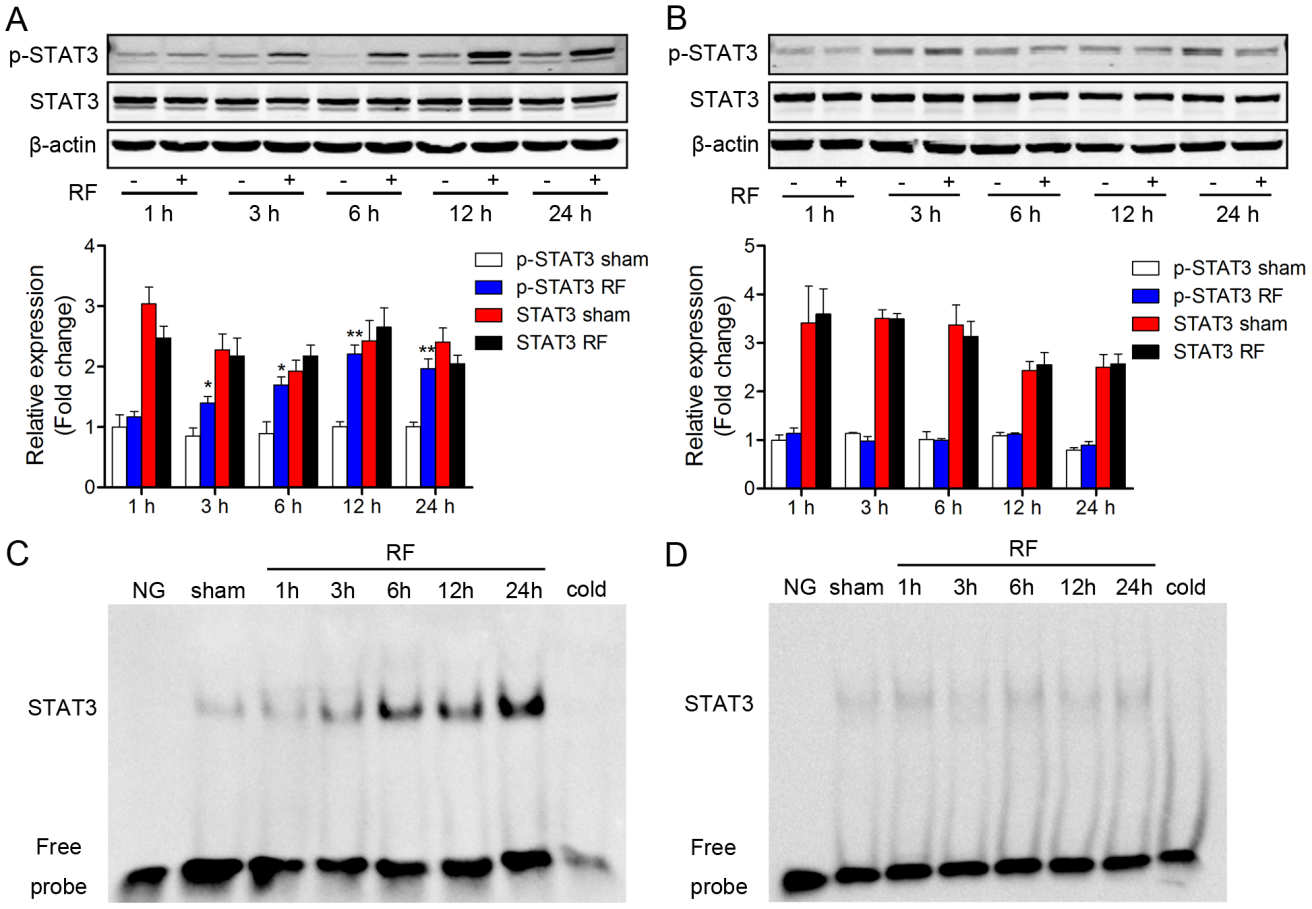
Expression, phosphorylation and DNA-binding activity of STAT3 after RF exposure in microglia and astrocytes. (A) Western blot assay for STAT3 expression and phosphorylation in microglial N9 cells. (B) Western blot assay for STAT3 expression and phosphorylation in C8-D1A cells. Upper panel: representative image of immunoblotting for total and phosphorylated STAT3 (p-STAT3) after RF exposure. Lower histogram: densitometric analysis of the western blotting bands. (C) EMSA assay for DNA-binding activity of STAT3 in N9 cells. (D) EMSA assay for DNA-binding activity of STAT3 in C8-D1A cells. NG: negative control. Unlabelled STAT3 probes were used as competitors at a 50-fold concentration. Values are mean ± SEM for three independent experiments. ^*^p<0.05, ^**^p<0.01 compared with corresponding sham groups.

The STAT3 signaling pathway has been suggested to play a critical role in the activation of microglia and astrocytes [31], [32]. To investigate whether the STAT3 signal is involved in 1800 MHz RF induced pro-inflammatory responses in both astrocytes and microglia, we detected the phosphorylation and DNA binding activity of STAT3 after RF exposure in both glial cell lines. Western blotting results revealed that phosphorylation levels of STAT3 (p-STAT3) significantly increased following 3, 6, 12 and 24-h RF exposure, despite the fact that the total levels of STAT3 were not altered at any time-point in microglia (Figure 6A). However, neither phosphorylated nor total STAT3 levels were altered by RF exposure at any indicated time-point in astrocytes (Figure 6B).

Phosphorylated STAT3 can translocate to the nucleus, binding specific DNA elements and regulating the transcriptional activity of target genes [36]. We performed EMSA to analyze the binding of STAT3 to DNA using a specific STAT3 probe. EMSA results showed that the binding capacity of STAT3 to DNA was enhanced by RF exposure in a time-dependent manner in microglia. STAT3 binding levels slightly elevated by 3-h RF exposure, dramatically increased after RF exposure for 6 h, and peak levels were reached at 24 h in microglia (Figure 6C). However, in astrocytes, DNA-binding of STAT3 remained low and was not affected by RF exposure (Figure 6D).

### STAT3 inhibitor attenuated pro-inflammatory responses induced by RF exposure in microglia but not in astrocytes

**Figure 7:**
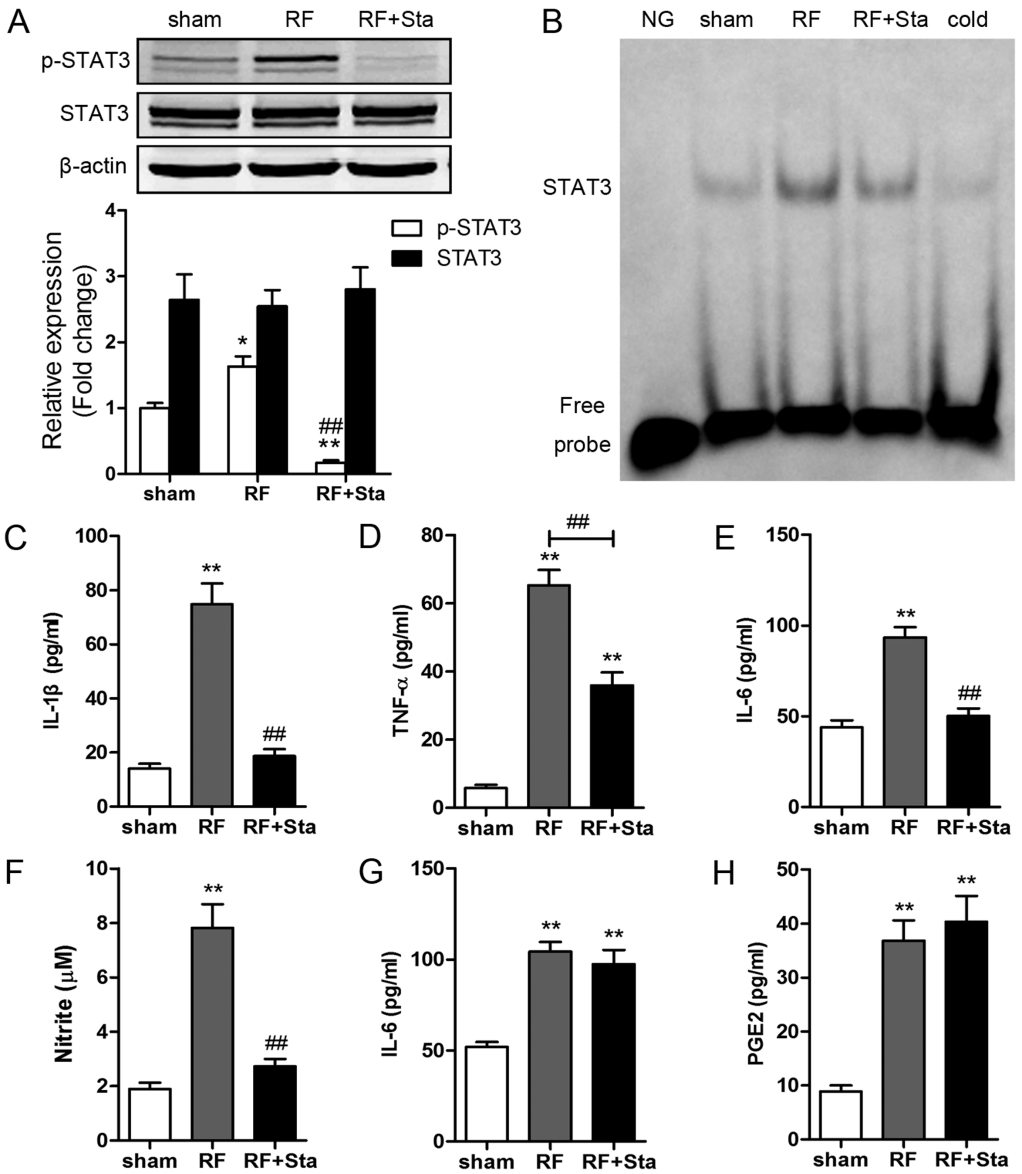
Stattic pretreatment inhibited pro-inflammatory responses induced by RF exposure in microglia but not in astrocytes. N9 and C8-D1A cells were pretreated with Stattic (Sta, 20 µmol/L) for 45 min, then exposed to RF for 24 h. (A) Western blotting result showing that RF-induced phosphorylation of STAT3 (p-STAT3) was blocked by Stattic in N9 cells. (B) EMSA result showing that Stattic pretreatment blocked RF-induced binding of STAT3 to DNA in N9 cells. NG: negative control. Stattic pretreatment inhibited RF-induced release of IL-1β (C), TNF-α (D), IL-6 (E) and NO (F) in N9 cells, but did not affect RF-induced release of IL-6 (G) and PGE2 (H) in C8-D1A cells. Values are mean ± SEM for three independent experiments. ^*^p<0.05, ^**^p<0.01 compared with sham groups, ^##^p<0.01 compared with RF groups.

To further assess the role of the STAT3 pathway in RF-induced activation of microglia and astrocytes, we explored the effects of Stattic, a specific STAT3 inhibitor, on RF-induced release of cytokines. First, we examined the effects of Stattic on the phosphorylation and DNA-binding activity of STAT3. Immunoblotting revealed that STAT3 phosphorylation (p-STAT3) induced by 24-h RF exposure was blocked by pretreatment with Stattic in microglia (Figure 7A). The EMSA results showed that Stattic completely blocked the RF-induced binding of STAT3 to DNA in microglia (Figure 7B). Moreover, STAT3 inhibitor attenuated the release of IL-1β, TNF-α, IL-6 and NO induced by 24-h RF exposure in microglia (Figure 7C, D, E, F). However, in astrocytes, Stattic pretreatment did not reduce the release of IL-6 and PGE2 after exposure to RF for 24 h (Figure 7G, H).

## Discussion

Astrocytes and microglia have been demonstrated to be differentially activated by a variety of stimuli, such as acrylonitrile, somatostatin, encephalomyelitis virus and spinal cord injury, presenting differential inflammatory response profile [37]–[40]. In the present study, both microglia and astrocytes were activated by RF exposure. However, RF exposure differentially triggered the expression and release of pro-inflammatory proteins and cytokines in microglia and astrocytes. A potential reason for this difference was provided by our subsequent experiments, which revealed that the phosphorylation and DNA-binding activity of STAT3 were up-regulated by RF exposure in microglia but not in astrocytes. Moreover, a STAT3 inhibitor ameliorates the RF-induced pro-inflammatory responses of microglia but has no influence on RF-stimulated astrocytes. Thus, our present study suggests that RF exposure can distinguishingly induce activation and pro-inflammatory responses in microglia and astrocytes, which involves differential activation of STAT3.

It has been previously suggested that activated microglia express various proteins and surface markers, of which CD11b has the greatest biological significance and is a typical feature of microglial activation [41]. Our previous study showed that microglial CD11b expression was increased by exposure to 2.45 GHz RF [17]. Consistently, the current study demonstrated that CD11b was up-regulated by RF exposure for 12 and 24 h in N9 microglial cells. This result indicates that exposure to GSM at 1800 MHz can potentially activate microglia, agreeing with previous reports that RF exposure activates microglia in vivo and in vitro [16], [17]. However, some other studies did not observe microglial activations after exposure to 900 and 1950 MHz RF fields [19], [42]. These conflicts might be due to the diverse RF signals with difference frequency, modulation, power density and exposure duration [43]. An intermittent exposure with 5 min on and 10 min off was used in our study, and this intermittent cycle has been reported to induce the greatest effects [44]. Further studies are needed to confirm the pro-inflammatory response triggered by 1800 MHz RF fields.

Astrocytes play an important role in maintaining brain homeostasis and limiting brain injury. Various CNS insults including traumatic injury, ischemia, neurotoxic chemicals, tumors and infections can cause astrocytes activation [25]. Astrocyte activation is characterized by increased expression of GFAP [45]. Our results showed that astroglial GFAP expression and immunoreactivity increased after 24-h RF exposure, indicating astrocytes activation. This result is supported by several previous studies. In animal models, acute and chronic exposure to RF have been reported to increase GFAP immunoreactivity in the brain [14], [15]. Similar to microglia, some studies did not observed astroglial GFAP reaction induced by RF fields under their modes [46], [47]. However, whether RF leads to gliosis is still a matter of debate. Some studies have demonstrated that the expression of cytokines can induce gliosis [48], and another study showed that pro-inflammatory cytokines were associated with radiation induced gliosis [49]. Furthermore, it is reasonable to attribute RF-induced GFAP to increased reactive oxygen species (ROS), considering that RF has been reported to induce ROS in astrocytes [50]. Additionally, GFAP expression has been shown to increase in response to oxidative stress [51].

Microglia play crucial roles in the process of neuroinflammation. Growing evidence suggests that microglia induced CNS injuries are mediated by the production of pro-inflammatory cytokines such as IL-1, IL-6, and TNF-α [21], [52]. Although the CCL2 were not affected by RF exposure, our results showed that RF exposure up-regulated the expression and release of IL-1β, IL-6 and TNF-α in microglial N9 cells. These results are consistent with previous reports that exposure to microwave increased the release and expression of TNF-α in peritoneal macrophages [53]. Additionally, IL-1β level has been found to be increased by 900 MHz RF electromagnetic fields exposure in the olfactory bulb in middle-aged rat [47]. We also found that RF exposure induced iNOS expression and NO production in microglial N9 cells. These results are consistent with our previous study, in which 2.45 GHz RF exposure increased release of TNF-α and NO, elevated iNOS mRNA in microglial N9 cells [17]. It has been also demonstrated that iNOS expression and NO production were up-regulated by low frequency electromagnetic fields in human monocytes and keratinocytes [54], [55]. Although RF exposure did not alter COX2 expression and PEG2 release, these results suggest that RF might facilitate microglial pro-inflammatory responses through the production of pro-inflammatory cytokines.

Astrocytes also participate in neuroinflammation by producing a variety of cytokines [45]. Thus, we detected the expression of astroglial cytokines after RF exposure, and a differential pro-inflammatory response profile from microglia was observed. The expression and production of IL-1β, TNF-α and CCL2 were not affected by RF exposure in astroglial C8-D1A cells. Also, iNOS expression and NO production did not differ after RF exposure. However, RF exposure increased COX2 expression and IL-6 and PGE2 release in C8-D1A cells. These results are coincidental with the RF-increased GFAP observed in our present study and some previous studies [14], [15]. Furthermore, our results are consistent with other studies in which RF was shown to cause apoptotic gene expression, ROS production, DNA fragmentation and apoptosis in astrocytes [3], [50], [56]. However, although RF frequency, power density and modulation are different, some other studies did not observe astrocyte response after exposures [18]. Thus, these controversial conclusions warrant further studies to confirm the effects of RF exposure on astrocytes.

Currently, few studies have focused on the mechanism of RF induced activation of microglia and astrocytes, especially the 1800 MHz RF. The STAT3 signal has been demonstrated to be involved in both astrocyte and microglia activation and drive the expression of inflammatory genes [31], [32], [57], [58]. The present study showed that phosphorylation of STAT3 was enhanced after 3-h RF exposure in microglial N9 cells. Moreover the DNA-binding of STAT3 increased with kinetics similar to phosphorylation of STAT3. These results suggest that STAT3 activation was invloved in the RF-induced microglial pro-inflammatory responses. Moreover, the STAT3 inhibitor Stattic effectively blocked or alleviated the release of IL-1β, TNF-α and IL-6 and ameliorated NO generation in microglial N9 cells activated by RF exposure. Thus these data demonstrated that STAT3 mediated the pro-inflammatory responses of microglia after RF exposure. However, in astrocytes, phosphorylation and DNA-binding of STAT3 were not altered by RF exposure, and RF-induced IL-6, COX2 and PGE2 were not affected by Stattic pretreatment. This result suggests that other signals might be involved in RF-induced astroglial responses, such as NF-κB, AP-1 and CREB which have been shown to mediate the inflammatory response in astrocytes [59], endowing astrocytes with a differentially pro-inflammatory phenotype compared with microglial responses mediated by STAT3.

However, in microglia, the RF exposure induced an expression of TNF-α at a same pace with STAT3 phosphorylation, and the STAT3 inhibitor incompletely blocked TNF-α release. A potential interpretation for these results possibly lies in that some other rapid pathway might activate microglia shortly after RF exposure. For example, NF-κB, AP-1 and C/EBPβ have been shown to mediate microglia activation and inflammatory factor release [60]–[62]. Previous study has revealed that the JAK2 inhibitor blocked delayed release of TNF-α and NO, but did not abolish the early TNF-α and NO release induced by 2.45 GHz RF exposure in microglia, indicating that other signaling pathways were involved in RF-induced microglia activation [17]. Moreover, STAT3 acts as a transcription factor regulating cytokine-induced pro-inflammatory responses [63]. TNF-α has been recently demonstrated to induce IL-6 expression via STAT3 pathway in C6 glioma cells [64]. Also, TNF-α treatment was reported to results in IL-1β release through STAT3 activation [65]. Therefore, it is reasonable to presume that TNF-α or other cytokines secreted by reactive microglia activated STAT3, which then induced and strengthened the expression of pro-inflammatory factors after RF exposure. And this hypothesis was supported by the present study, which showed RF exposure induced earlier TNF-α expression than other cytokines in microglia.

In summary, the present study demonstrates that 1800 MHz RF exposure differentially induced microglial and astroglial responses, which is partially mediated by differential activation of STAT3 in microglia and astrocytes. Although it is difficult to extend our result to human exposure, the data provide novel insights into the potential mechanism underlying the reported CNS impacts associated with mobile phone use and present STAT3 as a promising target to protect humans against increasing RF exposure. However, further studies have to be conducted to confirm these effects in vivo and adjust the results for real human exposure.
